# Patterns of lymph node metastases and their implications in individualized radiotherapeutic clinical target volume delineation of regional lymph nodes in patients with endometrial cancer

**DOI:** 10.7150/jca.78009

**Published:** 2022-10-31

**Authors:** Fei Teng, Hongfei Yu, Bin Wang, Ye Yan, Chao Gao, Fei Guo, Jinping Gao, Wenyan Tian, Yingmei Wang, Fengxia Xue

**Affiliations:** 1 Department of Gynecology and Obstetrics, Tianjin Medical University General Hospital, Tianjin, China.; 2 Tianjin Key Laboratory of Female Reproductive Health and Eugenics, Tianjin Medical University General Hospital, Tianjin, China.; 3 Department of Pharmacy, Tianjin Medical University General Hospital, Tianjin, China.

**Keywords:** Endometrial cancer, Lymph node metastasis, Radiotherapy, Clinical target volume

## Abstract

**Purpose:** To study the patterns of lymph node metastasis (LNM) of endometrial cancer (EC) and to clarify the individualized clinical target volume delineations of regional lymph nodes (CTVn).

**Methods:** Data from a total of 556 patients with EC who had undergone total hysterectomy associated with bilateral salpingo-oophorectomy (TH/BSO) and systematic lymphadenectomy were retrospectively examined. The clinicopathological factors related to LNM were analyzed using logistic regression analysis.

**Results:** LNM was found in 76 of 556 patients, resulting in a metastasis rate of 13.67%. The rates of LNM in patients with fundus and cornua lesions were 7.56% for para-aortic nodes and 14.41% for pelvic lymph nodes. The rates of LNM in patients with sidewall lesions were 3.15% for para-aortic nodes and 10.22% for pelvic lymph nodes. The rates of LNM in patients with lower uterine segment and cervix lesions were 12.50% for para-aortic nodes and 26.67% for pelvic lymph nodes. Deep myometrial invasion, histological type, histological differentiation, and lymphovascular space invasion (LVSI) emerged as statistically significant risk factors for pelvic LNM of EC (*P* = 0.008, 0.015, < 0.001, 0.005, respectively). Grade 3 differentiation had a strong influence on LNM to the para-aortic nodes (*P* = 0.015).

**Conclusions:** Myometrial invasion, histological type, histological differentiation, and LVSI were related to pelvic LNM and grade 3 was associated with para-aortic LNM. These factors should be considered comprehensively to design the CTVn for intensity-modulated radiation therapy (IMRT) of EC. For patients with lower uterine segment/cervix and fundus/cornua lesions, delineating the irradiation field of the para-aortic nodal region may confer a benefit.

## Introduction

Endometrial cancer (EC) is the fourth most common cancer among women in the United States; in 2021, it was responsible for 66,570 new cases of cancer and, with 12,940 deaths, was the sixth most frequent cause of death from cancer. Similar trends have been observed in China 2. Total hysterectomy associated with bilateral salpingo-oophorectomy (TH/BSO) and systematic lymphadenectomy are the standard treatments for EC. Postoperative radiotherapy is a routine component of multimodality treatments for patients with adverse risk factors, reducing the risk of locoregional recurrence. Generally, patients with risk factors, such as positive lymph nodes, age ≥60 years, presence of lymphovascular space invasion (LVSI), depth of invasion to the outer half, and higher grade are regarded as being at a “high risk” of recurrence 3.

Since 2009, the International Federation of Gynecology and Obstetrics (FIGO) has revised its staging criteria, emphasizing that pelvic and para-aortic lymph node metastases are classified into IIIC1 and IIIC2. Lymph node involvement is one of the most important prognostic factors and an indicator of the need for pre/postoperative radiotherapy. In recent years, intensity-modulated radiation therapy (IMRT) has reduced treatment-related toxicity and is becoming more widely available. IMRT integrates advances in engineering (multileaf collimators) and informatics (inverse dosimetric planning), which allows for the highly conformal delivery of therapeutic radiation doses to oncologic targets while minimizing the dose received by surrounding organs at risk (OARs) 4.

However, one of the factors limiting the widespread implementation of IMRT is the lack of a consensus on delineating the target volume. The clinical target volume (CTV) usually comprises the primary tumor or tumor bed and structures at risk of direct tumor spread, such as the draining lymph node regions. The pelvic and para-aortic lymph nodes are difficult to delineate, as most “normal size” lymph nodes are too small to be directly visualized with computed tomography (CT) or magnetic resonance imaging (MRI) but may still contain metastases. Therefore, it is necessary to include all lymph nodes within the draining regions of the tumor in the CTV. Accurate and reproducible clinical target volume delineation of regional lymph nodes (CTVn) is essential for effective IMRT. The study of risk factors related to lymph node metastasis (LNM) can help identify patients who are more likely to have involved lymph nodes and guide individualized radiotherapy. In the present study, we analyzed the pattern of LNM in patients who underwent TH/BSO and systematic lymphadenectomy in an effort to identify LNM-related predictors of CTVn delineation and to provide a basis for patient decisions regarding such delineation with or without surgery.

## Materials and methods

### Patients

We retrospectively studied the data of 556 patients with 2009 FIGO stage IA to IVB EC who attended the Department of Gynecology in Tianjin Medical University General Hospital from March 2003 to December 2018. The inclusion criteria were as follows: (a) patients with complete history, surgery data, histopathology report and physical examination on whom CT/X-ray of the chest, ultrasonography/CT of the abdomen, MRI/CT of the pelvic cavity, and/or positron emission tomography (PET)/CT had been performed for staging and evaluation of resectability of the primary tumor; (b) patients with a first diagnosis of histologically confirmed EC but no history of other malignancies or hormone therapy, radiotherapy, or chemotherapy prior to surgery; (c) the study was approved by the Ethics Committee of Tianjin Medical University General Hospital. The exclusion criteria were as follows: (a) patients with less than ten pelvic lymph nodes removed; (b) patients with unknown lymph node metastasis status; (c) patients with tumors diffusely spread throughout the uterine cavity. Clinical data were abstracted, including operative reports, pathology reports, and inpatient hospital records. Parameters related to the patients, including age, menopausal status, tumor locations, International Federation of Gynecology and Obstetrics (FIGO) stage, maximum diameter of the tumor, histological type, differentiation grade, lymphadenectomy, myometrial invasion, LVSI, and peritoneal cytology, were examined. Additionally, the number of regional lymph nodes examined and the number of regional lymph nodes with metastases were considered in the analysis.

### Surgical procedure and lymph node classification

All patients underwent TH/BSO, systematic pelvic lymphadenectomy and peritoneal washings by gynecologic oncologists. For patients with cervical invasion, extrafascial or radical hysterectomy was indicated. Omental biopsy was commonly performed in those with serous carcinoma or clear cell carcinoma. If extrauterine disease was suspected to be confined to the pelvic and abdominal cavities, surgical debulking was performed. Whether laparotomy or laparoscopic surgery was performed depended on the surgeon's preference - 470 (84.53%) patients underwent laparotomy, while 86 (15.47%) underwent laparoscopic surgery. The systematic pelvic lymphadenectomy included resection of the common iliac lymph nodes, internal iliac lymph nodes, external iliac lymph nodes, obturator lymph nodes and sacral lymph nodes. Adequate pelvic lymphadenectomy was defined as the removal of at least ten pelvic lymph nodes. Para-aortic lymphadenectomy was performed when the patients had high-risk factors, such as deep myometrial invasion, high-grade histology, serous carcinoma, and clear cell carcinoma, or when there were suspicious or enlarged para-aortic lymph nodes, as determined by intraoperative palpation. Resection of para-aortic lymph nodes involved lymph nodes located between the level of the aortic bifurcation and the level of the renal vessels. Dissected lymphatic tissues were placed in different specimen bottles according to their origin and sent for pathological evaluation.

### Pathologic analysis

All histological exams were performed and reviewed by pathologists subspecialized in gynecologic oncology for confirmation of the original diagnoses. The histological classification was performed according to the World Health Organization Classification of Tumors: Pathology and Genetics of Tumors of the Breast and Female Genital Organs (2014). Architectural grading was defined by standard FIGO criteria. All tumors were staged according to the FIGO 2009 staging system. The maximum diameter of the tumor, defined as the largest diameter of each of the three dimensions of the tumor, was measured on fresh tissue by pathologists. If more than 1 lesion was present, the lesion with the largest diameter was considered. The tumor locations were subdivided into the following categories: fundus and cornua (fundus/cornua), sidewall, and lower uterine segment and cervix (lower uterine segment/cervix). Fundus and cornua lesions were defined as the macroscopic presence of tumors in the fundus or cornua of the uterine corpus. The lower uterine segment was defined pathologically by the narrowest portion between the cervical os and the uterine fundus. On histology from these sections, pathologists can distinguish the junctional area of mucosa that represents the zone between the endocervical mucinous glands and the endometrial glands. The tumor location was labeled “Upper” for tumors of the fundus/cornua. The tumor location was labeled “Lower” for tumors of the lower uterine segment/cervix. The tumor location was labeled “Mid-corpus” for tumors of the sidewall. Histopathology reports stating involvement of the “endometrium” were coded with these three locations. LVSI was considered to be present when tumor cells were observed within or attached to the wall of a blood vessel or within the lymphatic space. LVSI was recorded as positive when this finding was revealed on H&E-stained sections. The number of lymph nodes was evaluated and recorded by two experienced gynecological pathologists.

### Statistics

The clinicopathological factors likely to influence the LNM rate (the number of LN-positive patients/the number of patients with involved LNs detected) and the LN ratio (ratio of metastatic to examined lymph nodes) of EC, including age, menopausal status, tumor location, maximum tumor diameter, histological type, differentiation grade, myometrial invasion, LVSI, and peritoneal cytology, were entered into the statistical analysis. All parameters were analyzed with respect to their relationship with LNM of EC using univariate logistic analysis. For multivariate analysis, the forward stepwise procedure was performed using a logistic regression model containing all variables in univariate analysis. All statistical tests were two-sided, and *P* < 0.05 was considered statistically significant. Analyses were performed using the SPSS 18.0 package.

## Results

### Patients and clinicopathologic features

A total of 556 patients were included in the present study. The median age was 58 years (range 28-78 years). There were 160 premenopausal patients (28.8%) and 396 postmenopausal patients (71.2%). Primary tumors were located in the fundus/cornua in 222 patients (39.9%), in the sidewall in 274 patients (49.3%), and in the lower uterine segment/cervix in 60 patients (10.8%). In terms of the FIGO stage, 341 patients were stage IA, 71 patients were stage IB, 32 patients were stage II, 15 patients were stage IIIA, 14 patients were stage IIIB, 71 patients were stage IIIC, and 12 patients were stage IV. The median maximum tumor diameter was 3.5 cm (range 0.5-11.0 cm). The maximum tumor diameter was ≤3.5 cm in 328 patients and >3.5 cm in 228 patients. There were 480 (86.3%) endometrioid adenocarcinomas, 25 (4.5%) papillary serous carcinomas, 10 (1.8%) clear cell carcinomas, 33 (6.0%) mixed carcinomas, and 8 (1.4%) other types of malignancies. In terms of the degree of differentiation, there were 282 patients with grade 1, 190 patients with grade 2, 54 patients with grade 3, and 30 patients of unknown status. Among the cohort, 278 patients (50.0%) had undergone pelvic and para-aortic lymph node dissection, and 278 patients (50.0%) had undergone only pelvic lymphadenectomy. The clinical and pathological characteristics of all 556 patients are shown in Table 1.

### Overall pattern of lymph node metastases

**Figure 1:**
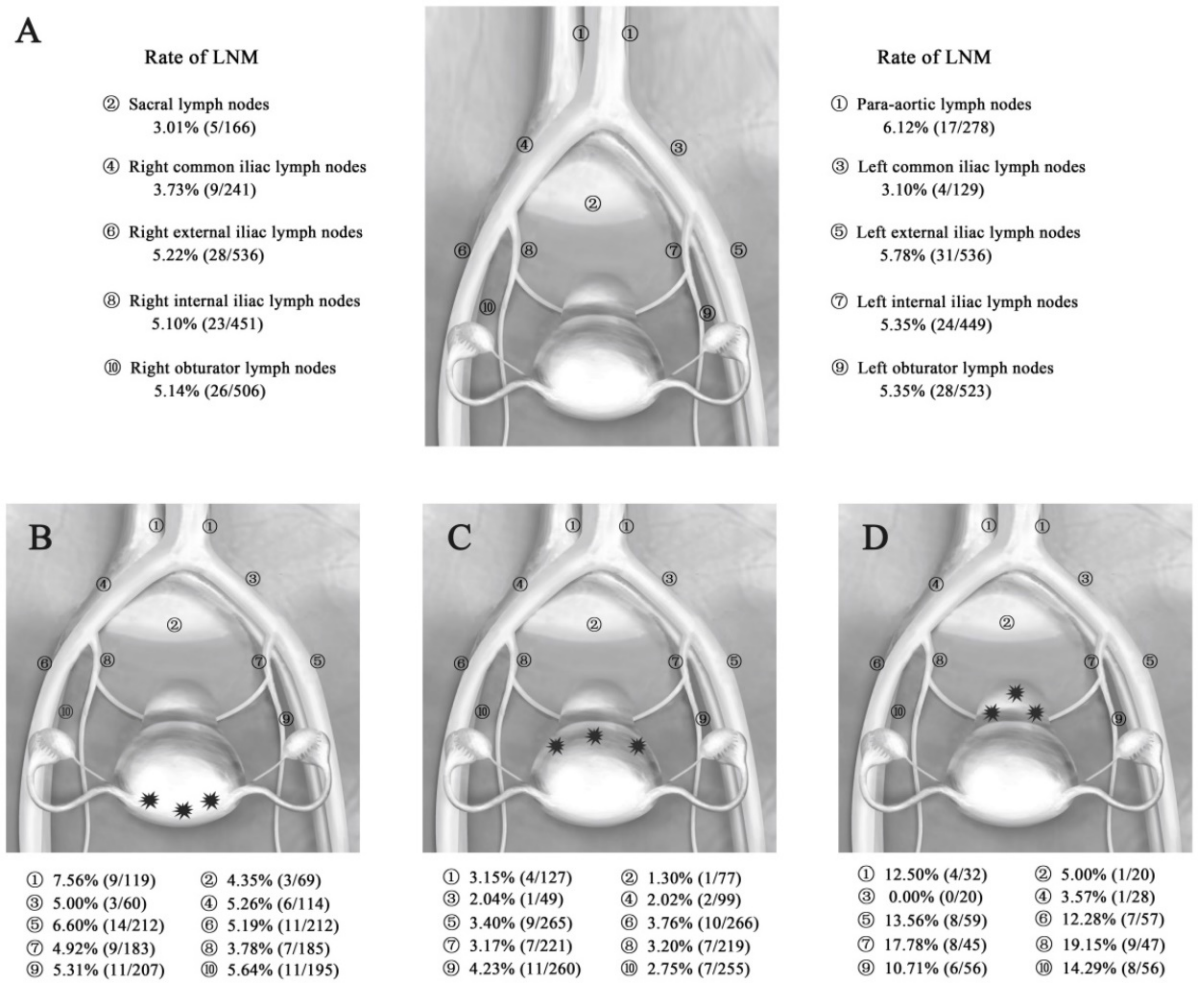
** Rate of LNM in EC patients. (A)** Rate of LNM according to the total primary tumor in 556 patients with EC. **(B-D)** Rate of LNM according to the different locations of the primary tumor in 556 patients with EC. **Abbreviations:** EC, endometrial cancer; LNM, lymph node metastasis. A, Rate of LNM for all patients with EC. B, Rate of LNM for EC patients with fundus/cornua lesions. C, Rate of LNM for EC patients with sidewall lesions. D, Rate of LNM for EC patients with lower uterine segment/cervix lesions.

A total of 10,408 lymph nodes were resected in all groups, and the mean number of dissected lymph nodes was 18.72 per person, with a range of 10-51. Lymph node metastases were found in 76 of the 556 patients; the rate of LNM was 13.67% (76/556), while the LN ratio was 2.68% (279/10,408). Fifty-nine patients had involvement of the pelvic lymph nodes, while one patient had only para-aortic lymph node involvement, and another 16 patients had both pelvic and para-aortic lymph node involvement. The incidence of pelvic LNM and para-aortic LNM was 13.49% and 6.12%, respectively. The most common site for pelvic LNM was the external iliac nodes (5.78%, 5.22%), followed by the obturator (5.35%, 5.14%), internal iliac nodes (5.35%, 5.10%), common iliac nodes (3.10%, 3.73%), and sacral nodes (3.01%). The proportion with metastases to the para-aortic nodes (6.12%; 17 of 278 patients) was relatively high. The rates of LNM by the different sites of EC are shown in Figure 1A.

### Tumor location and lymph node metastases

The total LNM rates in patients with lesions in different locations (fundus/cornua, sidewall, and lower uterine segment/cervix) were 14.41% (32/222), 10.22% (28/274), and 26.67% (16/60), respectively. The pelvic LNM rates were the same as the total LNM rates. The para-aortic LNM rates in patients with lesions in different locations (fundus/cornua, sidewall, and lower uterine segment/cervix) were 7.56% (9/119), 3.15% (4/127), and 12.50% (4/32), respectively. The total LN ratios for these different locations were 2.97% (127/4283), 1.40% (69/4937), and 6.99% (83/1188), respectively. Among them, the pelvic LN ratios for the different locations were 2.92% (110/3674), 1.51% (65/4299), and 7.00% (72/1028), while the para-aortic LN ratios for the different locations were 3.28% (17/519), 0.63% (4/638), and 6.88% (11/160), respectively (Table 2). Thus, the LNMs were more likely to be involved in patients with lower uterine segment/cervix lesions, while patients with sidewall lesions had LNMs that were relatively less involved.

The rates of LNM in different sites according to the location of the primary tumor are shown in Figure 1B-D. In the EC patients with fundus/cornua lesions, the most common site for LNM was the para-aortic nodes (7.56%), followed by the external iliac (6.60%, 5.19%), obturator (5.31%, 5.64%), common iliac (5.00%, 5.26%), internal iliac nodes (4.93%, 3.78%) and sacral nodes (4.35%). The rates of LNM in patients with sidewall lesions were lower: 3.15% para-aortic nodes, 1.30% sacral nodes, 2.04% and 2.02% common iliac nodes, 3.40% and 3.76% external iliac nodes, 3.17% and 3.20% internal iliac nodes, and 4.23% and 2.75% obturator nodes. In contrast, in patients with lower uterine segment and cervical lesions, metastases were detected in all sites with a higher frequency of LNM, especially in the internal iliac (17.78%, 19.15%), external iliac (13.56%, 12.28%), obturator (10.71%, 14.29%), and para-aortic nodes (12.50%). Generally, the most common metastatic sites of pelvic lymph nodes were the external iliac, internal iliac nodes and obturator nodes in EC. However, positive para-aortic lymph nodes often occurred for primary tumor of the lower uterine segment/cervix and fundus/cornua.

### Factors associated with pelvic and para-aortic lymph node metastases

Eight of the 9 clinicopathological factors, including age, tumor location, maximum diameter, myometrial invasion, histological type, degree of differentiation, LVSI, and adnexal involvement, showed obviously significant correlations with the pelvic LNM rates by univariate analysis (Table 3). The myometrial invasion, histological type, degree of differentiation, and LVSI were closely associated with the rates of para-aortic LNM by univariate analysis (Table 3). Although a significant difference between different locations of the uterine lesions in terms of the rates of para-aortic LNM (*P* = 0.057) was not detected, our study showed an orderly spread to the nodes at different sites that was clearly related to the different positions of the primary tumor. The relationships between LNM rates and various clinical characteristics are shown in Table 4, the multivariate logistic regression analysis revealed that pelvic LNM were significantly associated with myometrial invasion, histological type, degree of differentiation, and LVSI (*P* = 0.008, 0.015, < 0.001, 0.005, respectively; OR = 2.350, 2.701, 6.299, 4.128, respectively; 95% CI = 1.255-4.399, 1.212-6.017, 1.959-8.507, 2.500-15.867, 1.548-11.010, respectively). However, para-aortic LNM was strongly associated only with grade 3 differentiation (*P* = 0.015; OR = 7.323; 95% CI = 1.466-36.588).

## Discussion

EC is the most prevalent gynecologic malignancy in developed countries, affecting predominantly postmenopausal women of approximately 60 years of age. Its incidence has increased over time, especially in countries with rapid socioeconomic transitions. More than two-thirds of EC patients are diagnosed with stage I disease (usually detected early because of vaginal bleeding) 5, 6, for which the 5-year survival rate has been reported to range from 74 to 95%. However, metastasis is related to a worse outcome and for patients with stage III (accounting for 8.9%-20%) and IV (accounting for 3.3%-9%) disease 5, 7, 8, the 5-year survival rates decrease dramatically and range from 21 to 56% 9. Current postoperative treatment recommendations for advanced-stage EC consist of chemotherapy, radiotherapy or combined-modality therapy. Hogberg et al. 10 evaluated two protocols that randomized patients with high-risk EC to sequential WPRT and chemotherapy versus WPRT alone and showed a significant improvement in progression-free survival and disease-specific survival. Nevertheless, several studies 11-13 have shown that patients who received combined adjuvant chemoradiotherapy had consistently better outcomes than patients who received WPRT alone, but they also suffered from persistent diarrhea, bladder irritability, vesico-vaginal fistula, rectovaginal fistula, and pelvic fibrosis. Considering the treatment-related adverse events for WPRT, other more advanced radiation therapy techniques have been introduced. IMRT enables delivery of the prescribed dose to at-risk targets while limiting the dose to adjacent normal tissues. Chen et al. 14 published the following results from studying EC patients treated with IMRT versus conventional radiotherapy: Grade 2 acute gastrointestinal symptoms were reduced from 55.6% to 27.7%, and late gastrointestinal toxicity decreased from 19.4% to 3.1%. It is better to apply IMRT combined with chemotherapy to reduce complications and improve the local control rates of EC patients. The key to maximizing the efficacy of IMRT is to accurately and reproducibly delineate the CTV. The pattern of LNM is one of the major factors influencing OS, and it also affects the therapeutic decision-making for EC. However, there are currently no consensus guidelines based on risk factors derived from clinicopathological and surgical information for the optimal delineation of radiotherapeutic CTVn of IMRT for EC patients. In the present study, we aimed to demonstrate the precise mapping of LNM in EC patients who underwent systematic lymphadenectomy and to obtain useful information on how to define individualized CTVn for patients with EC who are slated to undergo IMRT.

The tumor locations in our study were recorded in the histopathology reports. There was significant difference between different locations of the primary tumor in terms of the rate of pelvic LNM and showed an orderly spread of lymph nodes related to the location of the primary tumor (Figure 1B-D). Based on our results, it is very important to accurately assess tumor locations in EC patients before surgery. At present, magnetic resonance imaging (MRI) is recommended by the International Federation of Gynecology and Obstetrics (FIGO) and the National Comprehensive Cancer Network (NCCN) as a preoperative diagnostic method for the assessment of local extent in EC 15. Similarly, the European Society of Urogenital Radiology also recommends the addition of tumor location in MRI reports 16. In parallel, transvaginal ultrasound is excellent in determining cervical stromal involvement, focal endometrial thickening, irregular endometrial margins, and a polypoid mass in the endometrial cavity. As a reproducible, dynamic, and noninvasive examination, the routine application of transvaginal ultrasound in EC is recommended. In addition to MRI and ultrasound, hysteroscopy can be a useful method in the examination of the tumor location of EC when the tumor is confined to the uterine corpus. Since the true positive rate is not high and it is an invasive procedure, hysteroscopy should be performed with restrictions.

The uterus has a rich lymphatic drainage network, and LNM may present as regional metastasis, distant metastasis, or skipping metastasis. In theory, lymphatic dissemination of EC fundamentally depends on the site of the primary tumor, but it is more complex because it may follow several drainage pathways while associating with one predominant pathway 17. In our study, compared to patients with EC tumors located in the fundus/cornua and sidewall, patients with lesions located in the lower uterine segment/cervix had much higher rates of pelvic and para-aortic LNM (Table 2). Tumors in the fundus/cornua of the uterus are often more likely to migrate to higher levels through the hypogastric route to the junctional lymph nodes (located at the junction between the internal and external iliac vessels) and then toward the common iliac lymph nodes and posteriorly to the para-aortic lymph nodes 18, 19. Therefore, the most common metastatic site in our fundus/cornua group was the para-aortic nodes, followed by the external iliac, obturator, common iliac, internal iliac nodes and sacral nodes, which is consistent with the canonical drainage routes in EC. Involvement of the lower uterine segment, cervix, or both is associated with an increased risk for LNM as well as recurrence 20, 21. The lesions located in the lower uterine segment/cervix had bidirectional metastasis trends, including regional metastasis to the pelvic lymph nodes and distant metastasis to the para-aortic lymph nodes. Their common drainage route is the lateral channel through the cervical stroma to the supraureteral, infraureteral, and neural paracervical pathways and then to the obturator lymph nodes and the medial chain of the external iliac lymph nodes, which are the most frequently affected 18. The posterior lymphatic trunk runs through the sacrouterine ligament and eventually drains into the sacral, common iliac and para-aortic lymph nodes. We considered that the rate of para-aortic LNM in EC patients with lower uterine segment/cervix invasion might be higher than that in early-stage cervical cancer (FIGO stage IA-IIA) patients, while the rate of pelvic LNM is comparable to that in patients with early-stage cervical cancer. Because the para-aortic LNM rate in stage IB1 cervical cancer patients is approximately 2%-5%, with the increase in the FIGO stage, the rate in stage II increases to 7%-17% 22. Skip metastasis means that the tumor achieves para-aortic LNM without pelvic LNM. Although skip metastasis is sometimes present, it is extremely rare; only one case occurred in this study, involving tumor that was poorly differentiated, large and deeply invasive. Direct spread to para-aortic lymph nodes via gonadal vessels is a possible route of lymphatic spread. In addition, aberrant and newly formed complicated lymphatic networks may result in more traffic branches directly connecting the primary tumor to para-aortic lymphatic areas 17, 23. Theoretically, radiation to a wider range of lymph nodes increases the chances for a cure. However, such radiation may not be beneficial to the prognosis if there are no lymph nodes affected. From the present study, we found that positive para-aortic lymph nodes often occurred when the primary tumor was in the lower uterine segment/cervix and fundus/cornua. Thus, we recommend delineating and enlarging the para-aortic nodal region when these two tumor locations are involved.

The present study showed that the rate of pelvic LNM of EC increased with higher histological type, myometrial invasion and LVSI, as well as decrease in tumor differentiation (*P* < 0.05). To make analysis more intuitive and convenient, the histological type of EC was classified as endometrioid and nonendometrioid EC. The special aggressive biological behavior of nonendometrioid EC means it is significantly associated with pelvic LNM and worse clinical outcomes. In accordance with previous studies 24,25, we also demonstrated that with increasing grade of differentiation, the rate of pelvic LNM gradually increased. Myometrial invasion is a much earlier molecular event and could be the initial driving force for the further progression of cancer cells. LVSI has been postulated as one of the first steps in the metastatic spread of EC and is accepted as a prerequisite for lymphatic dissemination 26. The likelihood of pelvic LNM increases dramatically with deeper myometrial invasion and LVSI, a clear reflection of increasing exposure to lymphatics as a tumor grows along and through the uterine wall 27, 28. Several studies, including the Gynecology Oncology Group (GOG) 99 and the PORTEC 1, 2 and 3 randomized trials, found that patients with positive LVSI treated with radiation therapy had a decreased risk of pelvic recurrence without significant improvement in OS 29-31. Based on these findings, the PORTEC Study Group recommended the consideration of adjuvant radiotherapy in EC patients with substantial LVSI, especially in the presence of additional risk factors 30. Therefore, it is important for a radiation oncologist to obtain this information before delineating the target volume. We suggest that the CTVn in EC patients with at least one of these clinicopathological factors should be correspondingly enlarged.

Li et al. reported that para-aortic lymph node involvement occurred with the highest frequency (6.6%) among all metastatic sites in a study, that included systematic pelvic and para-aortic lymphadenectomy in 4,001 patients; however, when pelvic lymph nodes are positive for carcinoma, the risk of para-aortic lymph node involvement rises to 47.3% 32. Patients with positive para-aortic lymph nodes were associated with higher all-cause and EC-specific mortality 33. Some physicians recommend that CTVn of IMRT should cover the para-aortic lymph node chain in women with FIGO stage IIIC1. However, there is a lack of consensus on the indications for para-aortic lymph node radiation, the extent of the radiation target volume, and the appropriate RT dose. In our study, we found that grade 3 was associated with para-aortic LNM. Therefore, when a tumor with grade 3 is indicated, we recommend including the para-aortic lymph nodes in the treatment volume.

According to the distribution pattern of LNM described above, personalized delineation of CTVn based on clinicopathological risk factors should be considered. The Radiation Therapy Oncology Group (RTOG 2008) 34 achieved a consensus guideline on CTVn definition for the postoperative treatment of EC. This international template of CTVn included the common iliac, external iliac, internal iliac, obturator, and presacral lymph node (in patients with cervical stromal invasion) regions. However, they mainly used bony landmarks to define the margins and did not include guidelines for para-aortic nodal volume delineation. Very recently, the NRG oncology and RTOG updated the previously described consensus guidelines 35. In this new consensus set of guidelines, the experts not only added the delineation of the para-aortic region for patients who required extended field radiation but also included the individual nodal groups that may be particularly difficult to define. However, for treatment planning of individual patients, these guidelines do not take into account the individual patient's anatomical variation and clinicopathological risk factors. The results of our subgroup analysis suggest that radiation oncologists should design individualized radiotherapeutic CTVns for EC patients with clinicopathologic and surgical information.

As a retrospective analysis, there were some limitations in the current study. First, this retrospective study led to selection bias. Moreover, during the 15-year study period, significant improvements in surgical techniques may have affected the results. Second, a single institution setting with a limited sample size could impact the statistical power, and the results are not representative of the whole population; thus, a multicenter study with a large sample size is needed. Third, not all cases underwent para-aortic lymphadenectomy during surgical staging. Fourth, the molecular classification of EC could play a role in prognosis and lymph node metastasis patterns, but due to national conditions in China, the lack of such data prevents us from making a more adequate assessment.

In conclusion, CTVn must be customized by experienced oncologists according to the risk factors that influence LNM. Irradiation of selected regional lymph nodes and their corresponding lymphatic drainage regions should be performed according to clinicopathological factors, such as deep myometrial invasion, nonendometrioid histology, LVSI and histological differentiation. For patients with lower uterine segment/cervix and fundus/cornua lesions, delineating the irradiation field of the para-aortic nodal region may confer a benefit. Our results can improve the accuracy of radiotherapy and provide a more individualized treatment for inoperable EC patients.

## Figures and Tables

**Table 1:** Clinicopathological features of 556 patients with endometrial cancer

Characteristics	Patients	
	Number of cases	Constituent ratio (%)
**Age**		
≤58	313	56.3
>58	243	43.7
**Menopausal status**		
Premenopause	160	28.8
Postmenopause	396	71.2
**Tumor location**		
Fundus/cornua	222	39.9
Side wall	274	49.3
Lower uterine segment/cervix	60	10.8
**FIGO stage**		
IA	341	61.3
IB	71	12.8
II	32	5.8
IIIA	15	2.7
IIIB	14	2.5
IIIC	71	12.8
IV	12	2.1
**Maximum diameter of tumor (cm)**		
≤3.5	328	59.0
>3.5	228	41.0
**Histological type**		
Endometrioid adenocarcinoma	480	86.3
Papillary serous carcinoma	25	4.5
Clear cell carcinoma	10	1.8
Mixed carcinoma	33	6.0
Other types	8	1.4
**Differentiation grade**		
Grade 1	282	50.7
Grade 2	190	34.2
Grade 3	54	9.7
Unknown	30	5.4
**Lymphadenectomy**		
Only pelvic	278	50.0
Pelvic + para-aortic	278	50.0

**Table 2:** LNM rate and LN ratio of different locations of the primary tumor (%)

Location	Rate of LNM (%)	Rate of pelvic LNM (%)	Rate of para-aortic LNM (%)	LN Ratio (%)	Pelvic LN Ratio (%)	Para-aortic LN Ratio (%)
Fundus/cornua	14.41(32/222)	14.41(32/222)	7.56(9/119)	2.97(127/4283)	2.92(110/3764)	3.28(17/519)
Side wall	10.22(28/274)	10.22(28/274)	3.15(4/127)	1.40(69/4937)	1.51(65/4299)	0.63(4/638)
Lower uterine Segment/cervix	26.67(16/60)	26.67(16/60)	12.50(4/32)	6.99(83/1188)	7.00(72/1028)	6.88(11/160)

LN, lymph nodes; LNM, lymph node metastasis; Rate of LNM, the ratio between the number of LN metastatic patients and the number of LN removed patients; LN ratio, the ratio between metastatic and examined lymph nodes.

**Table 3:** Univariate analysis of the clinicopathologic factors related to LNM

Characteristics	Pelvic LNM			Para-aortic LNM		
	Negative nodes	Positive nodes	*P* value	Negative nodes	Positive nodes	*P* value
**Age**			**0.040**			0.831
≤58	279	34		303	10	
>58	202	41		236	7	
**Tumor location**			**0.002**			0.057
Fundus/cornua	190	32		213	9	
Side wall	247	27		270	4	
Lower uterine Segment/cervix	44	16		56	4	
**Maximum diameter (cm)**			**0.020**			0.310
≤3.5	293	35		320	8	
>3.5	188	40		219	9	
**Myometrial invasion**			**<0.001**			**<0.001**
<1/2	382	32		408	6	
≥1/2	99	43		131	11	
**Histological type**			**<0.001**			**0.008**
Type I	431	49		469	11	
Type II	50	26		70	6	
**Differentiation grade**			**<0.001**			**<0.001**
Grade 1	271	11		279	3	
Grade 2	155	35		186	4	
Grade 3	35	19		47	7	
Unknown	22	8		27	3	
**LVSI**			**<0.001**			**<0.001**
Yes	14	17		26	5	
No	467	58		513	12	
**Peritoneal cytology**			0.794			0.935
Positive	59	10		67	2	
Negative	422	65		472	15	
**Adnexal involvement**			**<0.001**			0.391
Yes	21	16		35	2	
No	460	59		504	15	

*LNM*, lymph node metastasis; *LVSI*, lymphovascular space invasion. Bold text denotes statistical significance, *P*<0.05.

**Table 4:** Multivariate logistic regression analysis of the clinicopathologic factors related to rate of LNM

Characteristics	Pelvic LNM			Para-aortic LNM		
	OR	95%CI	*P* value	OR	95%CI	*P* value
**Age**	1.240	0.672-2.291	0.491	0.646	0.184-2.266	0.495
Tumor location						
Fundus/cornua	—	—	—	—	—	—
Side wall	0.675	0.354-1.286	0.232	0.429	0.117-1.575	0.202
Lower uterine Segment/cervix	1.042	0.421-2.578	0.929	0.723	0.143-3.653	0.695
Maximum diameter (cm)	1.320	0.718-2.426	0.372	1.754	0.521-5.906	0.364
Myometrial invasion	2.350	1.255-4.399	**0.008**	2.659	0.757-9.337	0.127
Histological type	2.701	1.212-6.017	**0.015**	1.256	0.262-6.022	0.775
**Differentiation grade**						
Grade 1	—	—	—	—	—	—
Grade 2	4.082	1.959-8.507	**<0.001**	1.627	0.346-7.658	0.538
Grade 3	6.299	2.500-15.867	**<0.001**	7.323	1.466-36.588	**0.015**
LVSI	4.128	1.548-11.010	**0.005**	2.610	0.456-14.944	0.281
Peritoneal cytology	1.011	0.410-2.498	0.980	1.672	0.317-8.810	0.544
Adnexal involvement	1.690	0.643-4.444	0.287	0.000	0.000-0.000	0.998

*LNM*, lymph node metastasis; *LVSI*, lymphovascular space invasion; *OR*, odds ratio; *CI*, confidence interval. Bold text denotes statistical significance, *P*<0.05.
